# What is the role of cultural competence in ethnic minority consumer engagement? An analysis in community healthcare

**DOI:** 10.1186/s12939-019-1104-1

**Published:** 2019-12-04

**Authors:** Reema Harrison, Merrilyn Walton, Ashfaq Chauhan, Elizabeth Manias, Upma Chitkara, Monika Latanik, Desiree Leone

**Affiliations:** 1 School of Public Health and Community Medicine, University of New South Wales, Kensington, 2052 Australia; 2 School of Public Health, University of Sydney, Sydney, New South Wales 2006 Australia; 3 School of Nursing and Midwifery, Deakin University, Geelong, Victoria 3025 Australia; 4 Honorary Professor, Melbourne School of Health Sciences, The University of Melbourne, Carlton, Victoria 3053 Australia; 5 Multicultural Health, Western Sydney Local Health District, Sydney, NSW 2151 Australia

**Keywords:** Cultural competence, Healthcare quality, Patient engagement, Healthcare consumers

## Abstract

**Background:**

Effective patient engagement has been associated with high quality health care. There is a dearth of evidence around effective engagement with consumers from ethnic minority backgrounds; specifically in relation to the role of cultural competence amongst healthcare professionals in effective engagement with consumers from ethnic minority backgrounds. To address this knowledge gap, we analysed the role of cultural competence in the consumer engagement approaches taken by community healthcare professionals working with consumers from ethnic minority backgrounds.

**Methods:**

Semi-structured individual interviews were conducted with 21 healthcare professionals employed across four community healthcare and affiliated services in four local government areas in Australia.

**Results:**

Adopting patient-centric approaches (that seek to understand and be responsive to the patient as an individual) featured as an underpinning theme that transcended other emerging themes. Recognition of diversity within communities and individuals in those communities, all with their own story, was described as pivotal to effective engagement. This was encapsulated in the theme of *Cultural standpoints and personal context* that contained four further themes of: (1) *Build foundations of trust and respect;* (2) *Diversify communication channels;* (3) *Generate system, service and community partnerships;* (4) *Take the time*.

**Conclusion:**

Our findings indicate that cultural competence and effective consumer engagement are closely linked in ethnic minority populations. Embedding cultural competence as a health system, service and professional capability is therefore critical to ensure equitable healthcare quality for consumers from all ethnic backgrounds.

## Introduction

Effective patient engagement is a feature of high quality health care internationally [1] that can be conceptualised on a spectrum from information exchange through to partnership. In these partnerships, patients contribute to decision making as they are considered a member of the healthcare team [2, 3]. Effective engagement can enhance healthcare quality in many ways such as taking into account information about patients’ beliefs, values and preferences. The ability to develop an understanding of patients’ beliefs, values and preferences underpins patient-centred care by supporting decision-making relating to screening, treatment or care options [4]. Achieving strong consumer engagement is therefore critical to patient-centric care.

The evidence indicates that consumers from ethnic minority backgrounds face a range of obstacles when engaging with health care services, that compound existing healthcare inequities [5, 6]. Having an ethnic minority background is associated with vulnerability to poor healthcare quality, and specifically the occurrence of safety events [7, 8]. Yet those from ethnic minority backgrounds are not well-represented in consumer engagement efforts that, in turn, negatively impacts the provision of patient-centric care for ethnic minority populations [9].

Cultural competence for healthcare professionals is defined as ‘the ability of healthcare providers to effectively deliver healthcare that meets the social, cultural and linguistic needs of their patients’ appears central in enhancing engagement of ethnic minority consumers [10]. Culturally competent healthcare provision is therefore central to realising the patient-centred care agenda for ethnic minority consumers. Cultural competence is a requirement consistently identified in health professional safety and quality frameworks [11, 12]. The Process of Cultural Competence Model (PCCM) considers cultural competence as the ongoing process in which health professionals continually seek to develop the skills that enable them to effectively work within the cultural context of the consumer. The process requires integration of cultural awareness, cultural knowledge, cultural skill, cultural encounters, and cultural desire [13]. ‘Cultural awareness’ has been described as the knowledge and in-depth examination and understanding on one’s own culture and how that shapes a perspective. The process of seeking out information about other cultural and ethnic groups including understandings and beliefs about health gives rise to the formation of ‘cultural knowledge.’ ‘Cultural skill’ and ‘cultural encounters’ in relation to health describe a professional’s ability to collect information from a patient and assess them in a cultural appropriate, and the direct cross-cultural interactions that occur between patient and professional. Finally, ‘cultural desire’ is the intrinsic motivation of healthcare professionals to engage in the process of developing the cultural competence [13].

Knowledge of how to apply cultural competencies when engaging ethnic minority healthcare consumers is lacking. To address this gap, our research analysed the role of cultural competence in the engagement efforts of community healthcare professionals working with ethnic minority communities in Australia. The study examined the experiences of healthcare professionals when engaging with patients in culturally and linguistically diverse communities, the approaches used to encourage patient engagement, and the elements of cultural competence that feature in their approaches. The emerging data were explored against the PCCM [13].

## Methodology

This research was conducted from a strengths-based perspective, grounded in appreciative inquiry [14, 15]. Data collection examined the existing approaches within the delivery of community health care and isolated the strengths to identify strategies that are or are perceived to be both positive and sustainable for engaging consumers from a range of cultural and linguistic backgrounds [16]. This research aimed to build on existing strengths to enhance quality of care within the cultural context of the consumer.

## Methods

### Ethical approval

Ethical approval was granted by the Western Sydney Local Health District Human Research Ethics Committee (HREC/17/WMEAD/499).

### Design

We used a cross-sectional qualitative descriptive study to capture experiences and perceptions of strategies to enhance engagement with consumers from a range of cultural and linguistic backgrounds. The study is reported in accordance with the Consolidated Criteria for Reporting Qualitative Studies (COREQ) guidelines to promote complete and transparent reporting of this focus group research [17].

### Setting

Community health centres in a public health district in Sydney were selected as research sites because the centre has a significant culturally and linguistically diverse consumer population. The selected health district has five acute hospitals, and seven community health centres, serving a population of approximately 946,000 residents [18]. Of the total health consumer population, 35% were born overseas or with one or both parents born overseas, and 43% speaking a language other than English, comprising language groups of Arabic, Mandarin, Dari and Turkish [18].

Community health centres provide services associated with the prevention, early intervention and community-based treatment, palliative care, child and family, counselling and rehabilitation services. We also included local district professionals providing oral health, mental health and youth health services [18]. Our research team also included two members from the district Multicultural Health Services. Multicultural Health Services work in collaboration with clinical, non-clinical and community partners and consumers with a remit to improve access to health services, empower diverse communities to actively participate in their healthcare and improve organisational capacity to respond to health needs of ethnic minority consumers and populations.

### Sample

Eligible participants were front line healthcare professionals and service managers with responsibility for policy development and implementation of health programs. Participants were purposively selected from across a range of services provided by the district and accessed by consumers from a range of age groups and health status. Invited services were: maternity, counselling, child and family health, youth, chronic disease and multicultural health services.

### Procedure

Given the geographic and service distribution of community healthcare professionals semi-structured interviews were selected as a flexible and suitable data collection method to obtain in- depth data with the flexibility to pursue other enquiries relating to the participants’ engagement strategies and techniques. Face-to- face or telephone interviews lasting between 30 and 50 min were audiotaped. The interview schedule included four topic areas [1]: perceptions and ability to effectively engage with consumers from different ethnic backgrounds [2]; approaches for engaging with consumers and address barriers to engagement [3], training designed for working with consumers and [4] perceptions regarding additional complexities associated with engagement with ethnic minority including refugee populations.

### Analytic strategy

Interviews were transcribed independently and then collectively analysed by a team of three researchers, including one health psychologist [RH], one medical doctor [UC] and one physiotherapist [AC]. The audio recordings were listened to repeatedly to become familiar with the data. Following transcription, each research team member independently conducted line-by-line coding, identified key words, phrases and sentences, and identified themes that developed from the data [19]. Coding was iterative and refinement of themes and subthemes evolved over the course of the analysis. A team-based approach to coding was used to establish inter-rater reliability [20]. Discrepancies were discussed and themes and subthemes refined until full agreement [21]. The data was then organised and displayed via diagrams and figures to identify patterns and interrelationships within the data. The analytic hierarchy comprised four levels of processing in which the researchers captured, described and explained the experiences and perspectives of each interviewee.

Measures were put in place to heighten the rigour of the analytic process. Ongoing discussion between the three primary researchers facilitated constant comparison, refining and defining themes and categories until a point of theoretical saturation. A further researcher [EM] then assessed the themes for face validity. The contribution of the wider research team in coding and categorisation checks, and discussion of the influence of the research context, promoted the credibility, confirmability and dependability of the analytic process [22].

## Results

A total of 21 interviews were conducted with health professionals and service managers from four district community health services across four local government areas (which can also be understood as councils/boroughs) in Sydney, Australia. Of the 21 participants, 19 were female with 2 males. The gender mix reflects the health professional profile of the district, in which women are in the majority. Nine participants were from a culturally or linguistically diverse background. Four themes emerged in addition to the following underpinning theme: Cultural standpoints and personal context. The four themes were: (1) Build foundations of trust and respect; (2) Diversify communication channels; (3) Generate system, service and community partnerships; (4) Take the time.

### Cultural standpoint and personal context

The adoption of patient-centric approaches that seek to understand and respond to the patient as an individual was an underpinning theme that transcended all other emerging themes. Recognising diversity within the communities and individuals in those communities, all with their own story, was pivotal to effective engagement. Being considerate and respectful of a consumer’s cultural standpoint and personal context featured as pre-requisites for effective individual engagement. Participants talked about the influence of a person’s cultural background and experience on how they perceived and interpreted their environment; their cultural standpoint in terms of what is socially acceptable and desirable behaviours/activities. They also identified the importance of personal context, which referred to the circumstances of an individual at any given time, such as exposure to recent trauma, their interpersonal relationship status and living arrangements; such circumstances may impact the individual’s priorities and feelings toward their health and healthcare.

*“..I do take each person individually, and if I know that the person I’m seeing, are planning to see, is from a different culture, I try to look a little bit, understand that culture before I see the person. Then in my introduction I do leave it open for the client to tell me … I do openly check with the person to say … is there anything else you want me to know about your culture? Or, is there anything that you would like me to do or not to do?” Counselling Services 1*

Cultural knowledge of the perspectives and expectations about the consumer-clinician relationship of each consumer and their community was a key ingredient. There was a sense that many consumers accessing the Australian health care system, had to shift from a system requiring them to be passive receivers of health-care to being engaged. Differing expectations could lead to mis-understandings about the nature of the care and of how to approach healthcare interactions. Knowledge of consumers’ level of trust in health professional expertise, perceptions about their role and the role of health professionals in decision-making, and the extent to which they might pose questions or raise concerns were all discussed as important foundational information from which health professionals might set expectations around engagement.

*“[this] is a new culture for them, everything new and they feel scared. They don’t feel confident. When they talking to you they feel like they can’t - you ask whether you have a question, they’re scared and that’s the culture they come from, that don’t ask any question for doctor or health profession. We say you can ask anything you want here.” Multicultural Health 2*

Personal context and prior experience for refugee patients, who were significant in numbers in the study district, raised additional issues. Being knowledgeable about the likely priorities, interests and thoughts held by a refugee patient was considered important. The quote below illustrates the health priorities amongst those who may have recently arrived in Australia following a traumatic period.

*“Like for someone coming here in Australia, starting from scratch and leaving all this drama behind, with some mental issues let’s say or trauma that they lived in, they will try to survive. Like survive I mean housing, I mean employment, I mean under-immunised kids. There are all these survival issues. So you wouldn’t go to them and talk first about, let’s say, let’s talk about your chronic disease and how you manage that, because they haven’t survived yet. So we need to focus on the importance - or prioritise what the needs are … you need to be mindful of that.” Multicultural Health 3*

Respect for different cultures and personal contexts is demonstrated by understanding disease incidence, lifestyle and health behaviours in each community. Long-held beliefs and practices often continue in a new country requiring careful navigation by health professionals. The complexity and challenges associated with consumer-centred and evidence-based health care relationships on issues such as tobacco use and weight management was described through the interactions described.

*“.. a big proportion of Egyptian men smoke, and they smoke a lot. And then, the healthcare practitioner advisors try to talk to them about the risks of smoking. So they really engrained belief that if they take ginger powder with lemon and hot water, their lungs can get better … .. I don’t think enough people know that as clinicians and practitioners, people not only bring a lot of their cultural beliefs, but they also bring their lifestyle and health behaviours from where they were born, before they migrated to Australia. They hold it really true to heart”. – Chronic Care Services 1*

Some health professionals from ethnic minority backgrounds raised the potential to better understand and empathise with consumers due to their own cultural background and awareness of the impacts this has on perceptions and behavours relating to health and healthcare.

*“So I know the culture of many countries because I lived with them, I grew up with - like here, in Australia. So my knowledge, my background knowledge helped me a lot with understanding different cultures and accepting different cultures and different beliefs, which is very helpful, really, for me. When I talk to the women, I know where they are coming from. “Maternity Services 3*

### Building trust amongst consumer communities

Trust in structures, resources and time comes through cultivating mutual respect. Participants saw trust as a fundamental characteristic for encouraging consumers to access and utilise healthcare. Maternal and youth health services were identified as critical avenues for establishing trust. Participants described some of their approaches for facilitating access for pregnant women and seeking to foster trust amongst young adults.

*“when you’re just sitting with them, you need to engage with them to make sure that you gain the trust from them … you have to put yourself in their position. Don’t look at them that you’re above with them … You start earning their confidence, wanting to come back to you”. – Maternity Services 2*

*“Now, to get them to first step I think a big barrier to people accessing the services is that the services are not known to those people, or are seen … as being a government service … if you’re government service they [can be] quite paranoid … so we need to deal with that, build that trust and knowing about who we are and the purpose of who we are, and the respect, it’s a process.” - Counselling Services 2*

### Generate system, service and community partnerships

Generating inter-sectorial partnerships were important for developing strong connections with patients. Effective relationships between the health system, health service and non-government organisations, including community-based and consumer organisations, were necessary for engagement opportunities. These partnerships were also valuable for up-skilling health professionals or for learning about their communities.

*“I think any building of relationships in the multicultural health area is really important, to just keep us informed and updated of new events or different initiatives that they’ve got” – Community Partnerships 1*

Cross-service collaboration within the participant’s district was particularly important for maternity liaison officers and family and child healthcare professionals who worked closely with the Migrant Resource Centre, drug and alcohol services for specific ethnic groups and charitable religious organisations.

*“We do a fair bit of work with specific NGOs. Some of the mosques and some of the mosque youth groups that we do some work with … Some of the work we’ve done with [service] a drug and alcohol program for African people...” Youth Health 1*

*“What we’ve done is we work in partnership. Even for a secondary settlement service or migrant resource organisation - health is one of the issues, but throughout partnerships with them we ensure that it’s one of the top three. We provide our services, our health education, through them in their context with a captive audience.” –Multicultural Health 2*

Notwithstanding policies for inter-sectoral collaboration, a disconnect between and within community and acute health services was raised as a missed opportunity for enhancing care quality and service engagement once patients access services. The opportunity for enhanced knowledge about a patient and their context when services speak to one another was highlighted.

*“So I think there’s also a bit of a disconnect between community health and what they do in the acute setting, and that’s why it’s good for us all to share information and actually collaborate a little bit more.” – Community Partnerships 1*

Closer connections between different health service groups facilitated optimal learning about ways to better work with consumers who came from other parts of the system and also to promote services to communities. Closer links could also identify and support community members presenting to a service but not be appropriately referred. The multicultural health service was seen as a key actor for connecting services, inter-sectorial organisations and communities.

*“we should have more partnership with those community multicultural services, the partnership with these services to help promote our service, but also to access to those clients from CALD background.” –Counselling Services 1*

Working with multicultural healthcare services within the district was important for developing culture specific programs and strategies for patient engagement. Specific examples included multicultural health workers from the service developing health programs or signposting support services to enable health professionals and patients to achieve their goals in health care provision.

*“when we first initially designed the [chronic disease management] program, we had a working group with the Multicultural Health unit, they reviewed the program and they looked at it to be how it could be applied across different cultural communities.” – Chronic Care Manager 1*

Four multicultural healthcare staff echoed the value of the Multicultural Health Service, for both reacting to queries and for reaching out to departments to improve programs for a range of different ethnic minority communities, often referred to as culturally and linguistically diverse (CALD) in the Australian context. The multicultural health team also reached out to technical colleges with enrolled refugees in English language classes to enhance aspects of health literacy that may support refugee consumers to identify health needs, negotiate the health system, and foster a connection to the health services available to them.

*“we work with individual service departments to improve their abilities to make the strategies that are relevant to CALD consumers. It’s either through engaging interpreters or working - engaging bilingual staff or working with communities - community organisations to make sure that we actually involve clients from CALD backgrounds.” - Multicultural Health 1*

*“We try to reach a bigger number of - especially refugees - we’re starting project with (Technical And Further Education colleges) TAFEs. When they actually learn English where we try to introduce some of the concepts … around some of the health issues which will then help them to engage in the health settings by using or at least understanding the concepts or using the language.” –Multicultural Health 1*

Front-line health professionals reported poor awareness of the opportunities offered by Multicultural Health. Proactive engagement from multicultural health workers with front line staff were identified as the main mechanisms to promote utilisation of multicultural health workers.

*“We don’t engage very well with Multicultural Health, so maybe it’s sitting there waiting for us, but we don’t use them. So we probably should have more to do with Multicultural Health services.” – Child & Family Health Nurse 1*

Developing partnerships with consumer groups was considered important for improving patient engagement in service planning and policy but participants perceived a gap in these partnerships. Key benefits of incorporating consumers at the system level included the development of programs that are appropriate and accessible, improving quality and safety by improving patient engagement, developing translated materials that are linguistically and culturally appropriate, and providing an avenue for gaining feedback on care provision and proposals.

*“I think one of the big barriers [to engagement] is CALD consumer representation on different working groups and even on the board.” – Maternity Services 1*

*“Consumer councils … [would enable] a stronger safety and quality message … having the right resources to know where to go and having a voice in the patient safety and quality area, is probably how, from my perspective, they would add a lot of value.”- Community Partnerships 1*

### Diversify communication channels

The need to move beyond well-established communication strategies (translated resources, interpreters and bilingual workers) was noted as important to facilitate effective communication about cultural beliefs, preferences and norms with a variety of communities.

Alternative approaches were presented predominantly by health professionals who conducted group programs or provided education sessions, with an emphasis on moving away from didactic education sessions. Engaging patients in practical hands-on activities, storytelling and videos were perceived to be more effective to communicate about and discuss health issues and services. The need to develop additional audio-visual materials (e.g. videos, pamphlets) for participants who are unable to read English or their own language was also identified.

*“I don’t know about other languages, but with the Arabic, I find people either they’re illiterate in their own language, or the language that they use is too classical Arabic, or the language that means many of these translation sometimes are not [congruent], are not appropriate as such...In my experience, I find people they respond more to phrases, short reading … plus pictures, or the visuals are much more effective..” –Counselling Services 2*

*“I don’t teach like I am an academic. It’s just a very simple word, work some activities with them and also have a bit of laugh or some [workshop] that lets them do what they can do and then give them some ideas.” – Maternity Services 2*

*“But you need to be having some practical things. Not only - use this translated material, because when they go home, they might not even read it.” – Maternity Services 2*

Health professionals also outlined approaches considered effective when working one-on-one with patients, using several engagement approaches to convey information. Some participants suggested that their approaches could be helpful if adopted on a larger scale, such as to capture patient-reported outcome measures.

*“So we use toys, we use [different] things, [unclear] and help her and her daughter to talk together through the toys, and help her to play out the situation through the toys” –Counselling Services 1*

*“But of course, verbal is not the only way to communicate with clients, and sometimes, even with adults, we use the drawings, try to elaborate the situation and feelings.” – Counselling Services 1*

### Take the time

Taking the time to build a relationship required multiple sessions with consumers and allowing adequate time for each session to enable challenges to be addressed, was a priority for most participants. Participants recognised this as a practical consideration when trying to engage with ethnic minority consumers. A consistent thread of findings converged that with time, it was possible to establish effective modes and styles of communication with consumers critical to forming a relationship. There was a strong sense of the desire to create successful relationships from which consumer partnership in care might form. This theme highlighted a practical requirement that complimented the wider activities identified by the participant group.

*“Sometimes it will take time, it will take more than one session for them to engage with the service provider, like with someone like me. So it’s not necessarily that they will open from the first time I see them. I may need to see them more than once.” – Maternity Services 3*

*“it’s usually double the length of a regular interview because I’m communicating small bits of information so the interpreter can communicate that to the patient and then the patient or the person is then - might have additional information tacked on to that bit of communication which comes back to me. So it’s like that’s a slower process.” – Chronic Care Nurse 1*

Participants reported that sessional time limits for certain programs and interpreters did not align with the needs of ethnic minority consumers. Developing relationships were best done without sessional limits.

*“We take the time to do that. We’re really into that long - being involved with people for the long haul. We don’t do short sessions. Like Community Health counselling, for instance, has a six, 12 session limit. We are unlimited.” – Youth Health 1*

Taking time to get to know an individual was an opportunity for health professionals to understand individual and intra-ethnic differences between consumers and how they could adapt their behaviour to accommodate individual preferences.

*“So you’re trying to get a feel, I suppose, trying to get a feel by asking them rather than guessing or expecting that we know. Because some people will say, well, this culture always believes this and this culture always believes that and they all do that and they all do that. Well, do they all? Let’s ask them first, ask what they need and what they want from us.” Family and Child Health Nurse 1*

## Discussion

Our findings suggest that features of cultural competence amongst healthcare professionals are closely linked to the methods health professionals use to engage consumers from ethnic minority backgrounds and the level of engagement they achieve. The methods described by participants included the development of a foundation of trust and respect with healthcare consumers through getting to know them, their ethnic background and what that means for their healthcare. These principles are consistent with the notion of getting to know patients as individuals that is at the heart of patient-centred care [3].

The need to consider health care provision more broadly than just the patient came through strongly in the reference to building relationships and trust at a community level and through inter-sectorial partnerships linking health and social well-being. This aligns with a broader movement in health systems around linking health and social care to facilitate a more holistic and person-centred approach [23].

The findings indicated that a sophisticated level of cultural competence amongst healthcare professionals contributes to the generation of ideas and techniques that may support effective engagement with ethnic minority consumers. Engagement strategies occurred at multiple levels from rapport and trust building in the lead-in to care, use of communication aids and extended session time during encounters, and inter-sectorial partnership to build capacity and the community networks that underpin high quality health care at a service and system level. These approaches are not novel, but appeared effective as part of a multi-modal approach to engaging communities from a range of backgrounds.

Exploring the findings in the context of each element of the Process of Cultural Competence Model (cultural awareness, knowledge, skill, encounters and desire), a strong cultural awareness among the study participants of their own cultural context and the implications of this for their practice emerged, particularly those from ethnic minority backgrounds [13]. The value of establishing cultural knowledge came through strongly. Cultural knowledge is described as the process of seeking and gathering a in-depth understanding of key belief areas relating to those from a range of backgrounds [13]. Knowledge of health-related beliefs and cultural values, disease incidence and prevalence, and beliefs regarding treatment efficacy are identified as critical in the model. It is well documented that such beliefs play a critical role in the healthcare behaviours and activities of different populations [24]. Evidence of this process emerges in a range of examples in the present study, such as the discussion relating to beliefs around smoking outlined by one participant.

While the notion of cultural skill was less visible in the context of research in community health and related services such as Youth Services, participants did discuss the application of specific programs to particular communities in relation to their health needs and vulnerabilities such as consideration of the application of a chronic disease program for a range of communities. Stronger examples emerged throughout the findings in relation to the concept of ‘cultural encounters.’ Participants invested significant energy and resources into their cross-cultural encounters to explore how to communicate effectively with particular individuals and groups, for example through the use of activities, toys and interpretation services. These findings reflect a significant body of research across health disciplines that focus primarily on cross-cultural communication [25–27].

The collective data emerging from the consumers in this study indicated a shared value in ‘cultural desire’ and that this was part of an underpinning belief system held by them. Cultural desire describes *‘a genuine passion to be open and flexible with others, to accept differences and build on similarities, and to be willing to learn from others as cultural informants.’* [28] Our findings indicate that cultural competence amongst healthcare professionals is closely related to the engagement of consumers from ethnic minority backgrounds and that central the value placed on cultural competence by healthcare professionals is central to the development of a culturally competent workforce.

### Implications

The development of cultural competence is a central feature of health professional educational frameworks, with interventions to enhance cultural competence therefore primarily emerging in the form of training and education of health professionals [12]. A systematic review of the effectiveness of cultural competence interventions in healthcare reported enhanced provider knowledge, attitudes, patient evaluations of care following health professional training but little evidence of the effectiveness of training alone on intermediate or treatment outcomes [29]. The review highlighted the effectiveness of culturally tailored interventions for treatment outcomes, such as a different duration or number of sessions and tailored communication techniques, that reflect many of the strategies described by our study participants [29]. These findings suggest that a multi-modal approach that incorporates strategies to facilitate engagement with ethnic minority consumers beyond the provision of translated brochures, such as with practical hands-on activities, storytelling and videos, may be valuable.

Addressing cultural competence at only the individual healthcare professional level has limited scope for enhancing ethnic minority consumer engagement across a health service or system. Our findings demonstrated the potential value of inter-sectorial partnerships and their importance for building cultural competence at a health and social care system level by supporting both staff and communities to develop cultural competence, and to understand how this may impact practice. Health systems and services may also consider specific issues at the service delivery level that have potential system impacts in terms of preventing hospitalisation through effective primary and community care, such as the enhanced opportunity for developing relationships with interpreters when no sessional limits are applied and how this may impact consumer health experiences and outcomes.

### Limitations

The inclusion of multiple community health services within the study provided perspectives from a range of different individuals, settings and service types, with broader principles for effective engagement with culturally and linguistically diverse communities emerging from these data. Yet, because our research was limited to a single metropolitan geographical district of Sydney, the findings may not be generalisable to other community health services nationally and internationally, or to rural and regional areas. The application of a strengths-based framework enabled us to address the research questions by examining positive and sustainable ways of working that demonstrate cultural competence. The use of such a framework however, does not account for health system and structural factors that contribute to the development of cultural competence in healthcare and to consumer experiences of engagement [30]. The project was focused to exploring engagement in relation to those from a range of cultural and linguistic backgrounds but further work may seek to delineate experiences of engagement amongst specific populations and individuals within.

## Conclusion

In the context of the value placed on consumer engagement for achieving high quality healthcare, the experiences of engagement among ethnic minority background consumers warrant further recognition and consideration. Our findings indicate that cultural competence and effective consumer engagement are closely linked in ethnic minority populations. Embedding cultural competence as a health system, service and professional capability is therefore critical to ensure equitable healthcare quality for consumers from all ethnic backgrounds.

## Data Availability

Please contact the authorship team to enquire regarding access to material.

## Abbreviations

CALD: Culturally and linguistically diverse
COREQ: Consolidated Criteria for Reporting Qualitative Studies
PCCM: Process of Cultural Competence Model
TAFE: Technical And Further Education Colleges
